# Latent Network Analysis of Executive Functions Across Development

**DOI:** 10.5334/joc.355

**Published:** 2024-04-02

**Authors:** Iris Menu, Grégoire Borst, Arnaud Cachia

**Affiliations:** 1 Department of Child & Adolescent Psychiatry, NYU Langone Health, New York, NY 10016, US; 2 Université Paris Cité, LaPsyDE, CNRS, F-75005, Paris, FR; 3 Institut Universitaire de France, Paris, FR; 4 Université Paris Cité, Imaging biomarkers for brain development and disorders, UMR INSERM 1266, GHU Paris Psychiatrie & Neurosciences, F-75005 Paris, FR

**Keywords:** Executive functions, development, network model, structural equation model, latent variable network model

## Abstract

Executive functions (EFs) are crucial for academic achievement, physical health, and mental well-being. Previous studies using structural equation models revealed EFs’ developmental organization, evolving from one factor in childhood to three factors in adults: inhibition, cognitive flexibility, and updating. Recent network model studies confirmed this differentiation from childhood to adulthood. Reanalyzing previously published data from 1019 children (aged 7.8 to 15.3; 50.4% female; 59.1% White, 15.0% Latinx, 14.3% Bi-racial, 6.7% African American, 4.2% Asian American, 0.6% Other), this study compared three analytical methods to explore EF development: structural equation model, network model, and the novel latent variable network model. All approaches supported fine-grained EF-specific trajectories and differentiation throughout development, with inhibition being central in childhood and updating in early adolescence.

A classical definition of executive functions (EFs) describes a set of high-level cognitive functions that enable individuals to intentionally regulate their thoughts and actions to successfully achieve their goals (Diamond, 2013). Since EFs are too broad to be modelized computationally or measured by a single variable, EFs have been either operationalized in a more precise manner or broken down to be studied (Nigg, 2017). Using structural equation modeling, Miyake et al. (2000) have laid the foundations in their model of EFs with inhibition, updating and shifting forming three distinct (*diversity of EFs*) but still correlated (*unity of EFs*) latent factors or constructs. Each EF would therefore be composed of a common part to all three EFs (*common-EF*) and a specific part to the EF in question (*EF-specific ability*; Miyake & Friedman, 2012). Inhibition or inhibitory control can be defined as the ability to resist automatisms and distractions in order to activate the appropriate response in conflict situations (Diamond, 2013). Updating or working memory updating allows us to keep information in memory and to perform operations on it (Diamond, 2013). While working memory refers to the ability to store and process information simultaneously (Engle et al., 1992; Oberauer et al., 2016), updating refers to the ability to operate on this temporarily stored information in the light of new incoming information and to update working memory regarding the results of this operation (Ecker et al., 2010). Finally, cognitive flexibility, also referred to as switching or shifting, is the third EF classically described: it allows one to switch between different instructions, strategies and thus to move from one cognitive operation to another (Diamond, 2013).

Of note, this distinction of three EFs is commonly accepted in adults but only one common EF factor, with poor separation among EF tasks, is typically observed during early childhood (e.g., Wiebe et al., 2008; Willoughby et al., 2012). EFs differentiate into the three distinct factors (inhibition, updating and cognitive flexibility) from late adolescence/early adulthood (for a review, see Karr et al., 2018). Research on EF organization primarily uses structural equation modeling (SEM), which includes confirmatory factor analyses that allow to test whether theoretical models with an *a priori* determined structure fit the experimental data. Such models can represent a latent, unobserved factor (e.g., inhibition) from the shared covariance between manifest, observed variables (e.g., scores on the Stroop, stop signal, and Mickey tasks) while addressing the issue of measure-specific error (Weston & Gore, 2006). Therefore, these models allow researchers to make inferences about psychological constructs that can be considered as more reliable because of the absence of measurement error (Flora & Flake, 2017; Goring et al., 2021). However, constructing these models requires a large sample size (at least 100 participants) and 3–4 indicators per construct (Marsh et al., 1998). In the case of EFs, this means proposing a battery composed of at least 9 to 12 tasks, which can be quite challenging in a longitudinal sample. Karr et al. (2018) used confirmatory factor analyses to test seven factorial models of EF structure derived from 17 previous SEM studies on two samples: one of children and adolescents (ages 8.33–14.41) and one of adults (ages 17.30–72.24). The analyses supported a one-factor (i.e., unidimensional model with no clear distinction between the different EFs) or two-factor (with shifting merged either to updating or inhibition) structure in the child/adolescent samples, and a nested-factor structure (i.e., one common EF factor and two specific factors for both updating and shifting) in the adult sample (Karr et al., 2018). This result, along with the extensive literature on factorial organization of EFs through development, support a differentiation or segregation of the different EF components with age. Should this hypothesis gain support over the idea of a more integrated development of EFs, it would suggest that children are developing a more refined and complex set of cognitive skills, which has implications for interventions tailored to specific EF components.

This differentiation through childhood, with increasing EF diversity with age, is also supported by EF studies using network models (Hartung et al., 2020; Karr et al., 2022; Menu et al., 2022; Younger et al., 2023). Network modeling, unlike SEM, allows for the study of relationships between variables without any *a priori* theoretical assumptions. It is an exploratory analysis in which each observed variable is represented by a node, and the relationship between these nodes are estimated and represented through edges (Epskamp et al., 2012). This approach is a complementary alternative to SEM. By estimating all associations, it allows to visualize the interconnected nature of all the data and investigate all sources of overlapping covariance among the measures. This is especially relevant in the context of EFs, where tasks often involve more than one EF and are therefore non-pure measures (Miyake et al., 2000). However, the accuracy of these models can be affected by the presence of measurement errors, unlike SEM (Goring et al., 2021). Network models can investigate the development of EF structure with age and specify which EFs become more central to general executive processing and have a greater influence on other EFs as age increases. Using network model on a cohort of children aged 7 to 15 years, Hartung et al. (2020) found that the connections between the different EF tasks remained stable with age, except for inhibition tasks. The inhibition tasks differentiated early from the other EF tasks, with very poor inter-EF connections (Hartung et al., 2020). Of note, the authors of this study used four components of EFs: inhibition, cognitive flexibility, updating and working memory. Working memory and updating were found to have strong interconnections, leading to ambiguous or redundant edges in the network, which can make it difficult to interpret the true underlying relationships between the variables of the model. Another cross-sectional study used network model to examine changes in EF organization from 3 to 85 years of age and reported a differentiation of EFs from childhood to adolescence and a dedifferentiation during young adulthood, which accentuates at older adulthood (Karr et al., 2022). Interestingly, a recent study using an accelerated longitudinal design network model also support differentiation of EFs and found that the observed organizational changes between ages 8 and 14 also occurred for each age group within a single year (Younger et al., 2023). A better understanding of the age-related changes of EF organization at these ages is critical. EFs are closely linked to many aspects of life (see Diamond, 2013 for review) including academic achievement and school-related processes such as language (e.g., Gooch et al., 2016), reading (e.g., Nouwens et al., 2021) or arithmetic (e.g., Roell et al., 2019). These processes develop during the middle childhood period and their associations vary among EFs, ages, and academic outcomes (for a recent meta-analysis, see Spiegel et al., 2021).

In this context, the aim of this study was to investigate the organization of the three core EFs (inhibition, cognitive flexibility and updating) across development by reanalyzing previously published data (Hartung et al., 2020) from the Texas Twin Project cohort (Harden et al., 2013), but using a different methodology. EF were first estimated using network models in order to investigate the links between the different EF tasks without any *a priori* on the EF structure. Then, seven latent variable models were constructed in line with the work of Karr et al. (2018) and Miyake et al. (2000) to compare different EF structures, adding to Hartung et al. (2020)’s work which only investigated a bifactorial model organization. Finally, we used the recently developed latent network models (Epskamp et al., 2017), combining network and latent variable models. In latent network models, the researcher *a priori* defines latent variables, and a network is estimated among these latent variables, while taking measurement error into account (Epskamp et al., 2017). This approach enables the exploratory examination of conditional independence relationships between latent variables without assuming directionality or causality, unlike in SEM (Epskamp et al., 2017). These models are very appealing in the context of EFs since they enable to explore the relationships between the various theoretically-defined constructs. To date, no study has used latent network model approach in the context of EFs. The combination of SEM, network model and latent network model, allowing for the comparison of different theoretical models, aims at deciphering the complex and dynamics organization of EFs from 8 to 14 years. This period is crucial for the development of EFs and school acquisitions, such as reading comprehension, mathematical abilities, and problem-solving. Overall, this study could not only enrich basic knowledge but also have implications for the timing, design and implementation of EF-based interventions.

## Methods

### Participants

This study reanalyzes previously published data (Hartung et al., 2020), using weighted covariances matrices (see below, **Weighting function** section). These data were drawn from 1019 participants (50.4% female) from the on-going Texas Twin Project (https://sites.la.utexas.edu/twinproject/; Harden et al., 2013; Hartung et al., 2020) including children in Grades 3 to 8 (10.79 ± 1.76 years, range = [7.8 and 15.3]). Children were recruited from school registers (Harden et al., 2013). There were 479 twin pairs, 19 triplet sets, and 1 quadruplet set. In terms of race/ethnicity, 59.1% of the sample identified as White, 15.0% as Latinx, 6.7% as African American, 4.2% as Asian American, 0.6% as another race or ethnicity, and 14.3% as multiple races or ethnicities (Hartung et al., 2020).

More details about the Texas Twin Project and this sample can be found in Harden et al. (2013) and Hartung et al. (2020).

### Evaluation of EFs

We included 9 variables analyzed in the previous network model study (Hartung et al., 2020) derived from several tasks that assessed different EFs: *Animal Stroop, Stop signal auditory*, and *Mickey* for inhibition; *Trail making, Plus-Minus*, and *Local Global* for cognitive flexibility; and *2-Back, Running memory*, and *Keeping track* for updating. We did not include the three measures of working memory (*Symmetry Span, Listen Recall* and *Digit Span Backward)* in order to (1) be consistent with most of the literature on executive functions organization, which includes inhibition, cognitive flexibility, and updating only (e.g., Karr et al., 2018; Miyake et al., 2000), and (2) avoid highly correlated nodes and ambiguous or redundant edges in the network and latent variable networks that could make it difficult to interpret the true underlying relationships between variables or factors. Of note, analyses with the 12 variables are available in **Figure S10** in **Supplemental Materials**.

The tasks and the dependent variables are briefly described in Table 1; a more detailed description can be found in Hartung et al. (2020). Table 1 in Hartung et al. (2020) provides descriptive statistics and reliability estimates.

**Table 1:** **Description of the executive function tasks**. This table is based on Table S1 in Hartung et al., 2020.


	TASK DESCRIPTION	DEPENDENT VARIABLE

*Inhibition*		

Animal Stroop (Wright et al., 2003)	Participants were asked to verbally identify animals from drawings based on their body. In the congruent condition, the face of the animal matched the body; in the incongruent condition, the face did not match the body; in the neutral condition, the face area was blank.	Mean reaction time cost for incongruent conditions relative to congruent and neutral conditions

Stop signal auditory (Verbruggen & Logan, 2008)	Participants were asked to indicate which way an arrow was pointing, but to inhibit their response if a tone (stop signal) sounded after the presentation of the arrow.	Stop signal reaction time, i.e., subtraction of the average stop signal delay (time between arrow and stop signal presentation) from the mean go reaction time

Mickey(Lee et al., 2013)	Participants were asked to indicate on which side of a computer screen a cartoon Mickey Mouse face appeared while ignoring any squares that flashed on-screen before. In the congruent condition, a square flashed on the same side of the Mickey; in the incongruent condition, a square flashed on the opposite side; in the neutral condition, squares flashed on both sides.	Mean reaction time cost for incongruent trials relative to congruent and neutral trials

*Cognitive flexibility*		

Trail making (Salthouse, 2011)	Participants were asked to connect circles containing numbers in numerical sequence and circles containing letters in alphabetical order. In the two simple conditions, only numbers or letters were presented. In the two alternating conditions, both numbers and letters were presented, and the participants had to connect the circles in an alternating sequence (numbers–letters: 1-A-2-B, etc. or letters–numbers: A-1-B-2, etc.).	Switch cost, i.e., the mean reaction time for alternating conditions relative to simple conditions

Plus-Minus(Miyake et al., 2000)	Participants were asked to complete simple addition and subtraction problems on paper. In the adding condition, participants had to add 1 to each provided number; in the subtracting condition, they had to subtract 1; in the alternating condition, they had to alternate between adding 1 and subtracting 1.	Switch cost, i.e., the mean reaction time for alternating conditions relative to simple conditions

Local Global (Miyake et al., 2000)	Participants had to identify letters and shapes composed of smaller letters and shapes. In the two local conditions, participants had to name the small constituent letters or shapes; in the two global conditions, they had to name the large overall letter or shape; in the alternating condition, they had to alternate between naming local and global letters or shapes.	Switch cost, i.e., the mean reaction time for alternating conditions relative to simple conditions

*Updating*		

2-Back(Jaeggi et al., 2010)	Participants were asked to watch a sequence of shapes and to indicate when the current shape matched the shape from two trials prior.	Total number of hits (correct responses) minus false alarms (responses incorrectly identified as matching)

Running memory (Broadway & Engle, 2010)	Participants were asked to watch a sequence of single letters and to recall the last *n* letter.	Total number of letters correctly recalled

Keeping track (Miyake et al., 2000)	Participants were asked to listen to words falling under four categories and to recall the most recent word from a given category.	Total number of words correctly recalled


### Weighting function

Rather than grouping participants based on the moderator range, here age, observations were weighted around focal points (i.e., specific values of the continuous moderator variable). This procedure is thought to be beneficial for age-group analyses because parameter estimates have been shown to be more informative and less distorted with respect to age differences (Hildebrandt et al., 2016). SEM but also network model and latent variable network model were estimated sequentially for each focal age point using weighted samples of observations (Hildebrandt et al., 2016).

Concretely, following the recommendations of Hildebrandt et al. (2009), a Gaussian kernel function was used to weight observations for each specified value of the age variable (also referred as ‘focal age point’). The largest weight was for the specified age point and observations further from this age point had decreasing weights (Hildebrandt et al., 2009, 2016). The focal age points ranged from 8.0 to 14.0 with 0.1 increments. In total, 61 weighted (co)variance matrices were obtained (Hartung et al., 2020). Of note, the original team that collected the data and performed the initial analysis used the weighting function (Hartung et al., 2020) and shared the resulting weighted covariance matrices to perform the current study.

### Network models

Network models were constructed for each of the 61 focal age points based on the (co)variance matrices of the 9 EF variables. 61 networks were thus estimated. Nodes of the network corresponded to the scores at the 9 EF measures, which were grouped in three EFs. For each focal age point, network was estimated and standard graph centrality indices (degree, closeness and expected influence) were calculated. We used network models to analyze the multiple relationships (edges) between the different EF tasks (nodes) while also examining how these relationships change from 8 to 14 years of age.

All statistical analyses were performed using R-statistical software, version 3.6.3 (R Core Team, 2021). Network models were constructed and visualized using the package *qgraph* version 1.6.9 (Epskamp et al., 2012).

### Latent variable models

To investigate the factorial structure of EFs through development, we built and tested the seven factorial latent models described in (Karr et al., 2018):

- *Unidimensional model*: one latent factor defined by the 9 EF variables (Common-EF)
- *Cognitive Flexibility-Updating Merged model*: two latent factors model, one referring to inhibition and one referring to cognitive flexibility and updating merged
- *Inhibition-Updating Merged model*: two latent factors model, one referring to cognitive flexibility and one referring to inhibition and updating merged
- *Inhibition-Cognitive Flexibility Merged model*: two latent factors model, one referring to updating and one referring to cognitive flexibility and inhibition merged
- *Three-Factor Model*: three latent factors model, each referring to one EF (inhibition, updating and cognitive flexibility)
- *Nested Factor Model*: three latent factors model, one referring to cognitive flexibility, another referring to updating and a last one defined by the 9 EF variables (Common-EF)
- *Bifactor Model*: combination of the Three-Factor and the Unidimensional models: 4 latent factors, three referring to each EF (inhibition, updating and cognitive flexibility) and a last one defined by the 9 EF variables (common-EF)

Each model was constructed and then fitted separately for each focal age point. To find out which model provides the best fit for each of the 61 age points, the fit indices (BIC, CFI, RMSEA, SRMR) were compared for each model and each focal age point. The respective factor loadings and details of the estimates for each model were then inspected.

The latent models were constructed and estimated using the package *lavaan* version 0.6–7 (Rosseel, 2012).

### Latent network models

Finally, for each focal age point, a latent variable network model derived from the classical three-factor model was constructed and estimated following the method described by Epskamp et al. (2017).

This latent variable network model allows to combine the two previous approaches by assessing the network of the latent variables (representing inhibition, updating and cognitive flexibility), and not of the tasks themselves. Of note, in the case of the unidimensional EF model, there is only one latent variable (common-EF), and therefore there is no possibility to create a latent variable network model (a network requires at least two nodes).

The latent variable network model was constructed and estimated using the package *lvnet* version 0.3.5 (Epskamp et al., 2017).

The code associated to this study is available on OSF at https://osf.io/2bzjc/. The (co)variances matrices are available upon request to the authors and with formal agreement of Prof. Harden & Tucker-Drob. This study was not preregistered.

## Results

### Network models

**Figure 1:**
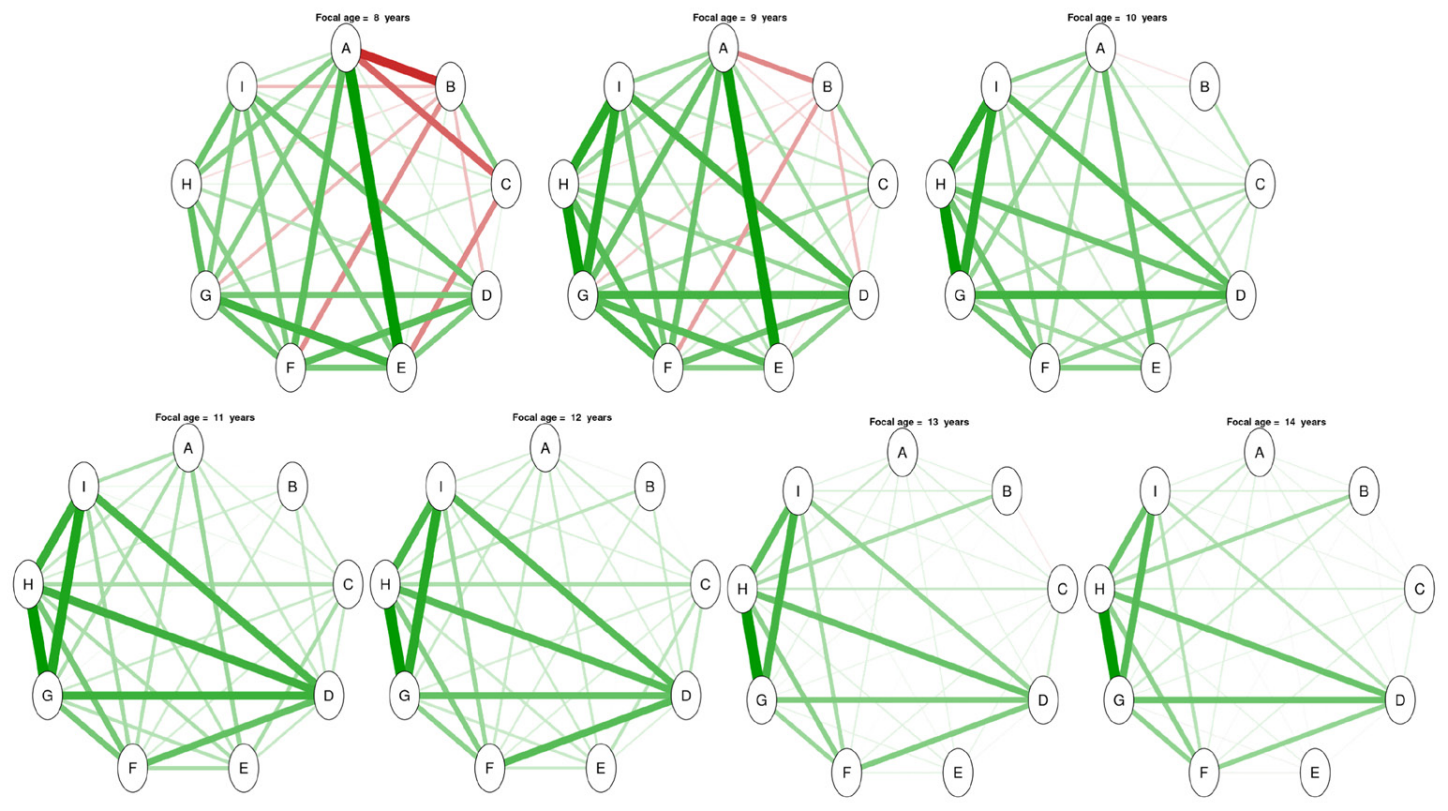
**Overview of the latent network model at different focal age points**. A = Stroop; B = Stop signal; C = Mickey; D = Trail making; E = Plus-minus; F = Local global; G = 2-back; H = Running memory; I = Keeping track. Green edges indicate positive weights, red edges indicate negative weights, and the thickness indicates the magnitude of these weights.

Network model analyses reveal a differentiation of EFs with age (see Figure 1 and online **Supplemental materials** for visual animations). While networks are initially very dense, with many intra- and inter-EF connections, their structure become sparser with age. In particular, all inhibition tasks are initially central in the network, with significant weight at the first focal age points, but quickly separate from the rest of the EF tasks. On the contrary, updating tasks gain more weight and become central with age.

These visual inspections were followed by a quantitative analysis of network topology using classical graph indices: degree, expected influence, and closeness (see **Appendix A1** and **Figures S1, S2**, and **S3** in **Supplemental Materials**), which further confirmed the observed differentiation process. For instance, the degree – the index of the number of connections for each node of the network, weighted by the size of these connections – of inhibition tasks diminishes drastically from 8 years of age while a strong increase of the degree of updating tasks is observed after 12 years of age (see **Figure S1** in **Supplemental Materials**). Similar results are observed regarding the closeness – the index of how strongly a node is indirectly connected with the network, calculated as the inverse of the total length of all the shortest paths between the selected node and all other nodes in the network – of inhibition tasks, suggesting that the indirect connections follow the same trajectories as the direct ones (see **Figure S3** in **Supplemental Materials**).

However, it is important to note that tasks within each EF do not follow exactly the same pattern with age. Tasks for inhibition, in particular, follow different trajectories. Indeed, the degree of Stroop is the highest at first and then decreases almost linearly with age until 12.5 y.o., preceding a slight rebound, whereas the degree of Mickey drops only until 9.2 y.o. before slowly increasing again, peaking at the focal age point of 10.2, and decreasing again slowly through 13.5 y.o. Therefore, the different inhibition tasks do not play the same role in the network through time. Similarly for cognitive flexibility, the graph indices of the different tasks also vary across ages. Of note, the situation is different for updating tasks which present high homogeneity with time.

Furthermore, the network analyses also show that tasks attributed to different EFs exhibited non negligible connections (i.e., shared non negligible variance). For instance, the edge linking Stroop (inhibition) and Plus-Minus (cognitive flexibility) tasks is among the most important edge in the network between 8 to 10 years and the edges between Trail making (cognitive flexibility) and all updating tasks are important throughout the entire age range.

### Latent models

In this section, we present results of the SEM analyses conducted on the same data with different EF structure and latent variables.

#### Model fit

Some models fail to converge at some focal age points. The bifactor model (i.e., the model that includes 4 latent factors, three referring to each EF and a fourth one defined by all 9 EF variables representing common-EF) only converges for a minority of focal age points (20 out of 61). The nested model (i.e., the model that includes three latent factors model, one referring to cognitive flexibility, another referring to updating and a last one defined by the 9 EF variables) fail to converge for 19 focal age points, mostly located at the endpoints (between 8 and 8.9 years and between 12.4 and 13.9 years).

**Figure 2:**
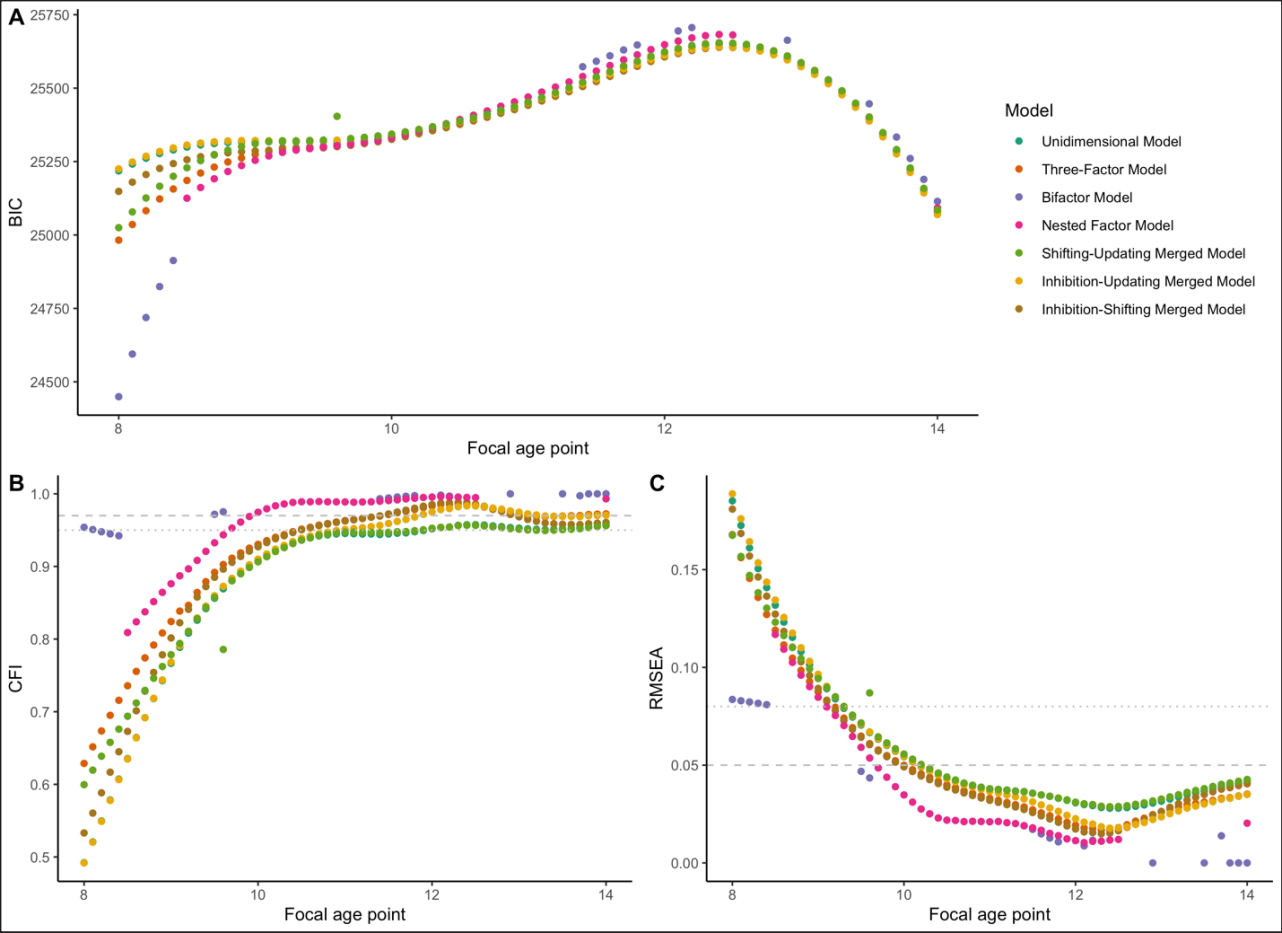
**Fit indices of the 7 latent models. (A)** = BIC (Bayesian information criterion) for each model by focal age point. **(B)** = CFI (comparative fit index) for each model by focal age point. **(C)** = RMSEA (root mean square error of approximation) for each model by focal age point. Dot line corresponds to threshold for acceptable fit (CFI > 0.95, RMSE < 0.08) and dashed line corresponds to threshold for good fit (CFI > 0.97, RMSE < 0.05; Schermelleh-Engel et al., 2003).

Regarding model fit measures (BIC, AIC, CFI), the bifactor model presents the best fit indices between 8 and 8.5 years, then, the nested model shows the best fit indices. However, fit variations between the different models are minimal and all models present correct fit (i.e., CFI > 0.95, RMSEA < 0.05; Schermelleh-Engel et al., 2003) after 10 years. Before this age, none of the models present acceptable fit (i.e., CFI < 0.95, RMSEA > 0.05). BIC, CFI, RMSEA fit results can be found in Figure 2. SRMR results can be found in **Figure S4** in **Supplemental Materials**.

#### Loadings of EF latent variables

**Figure 3:**
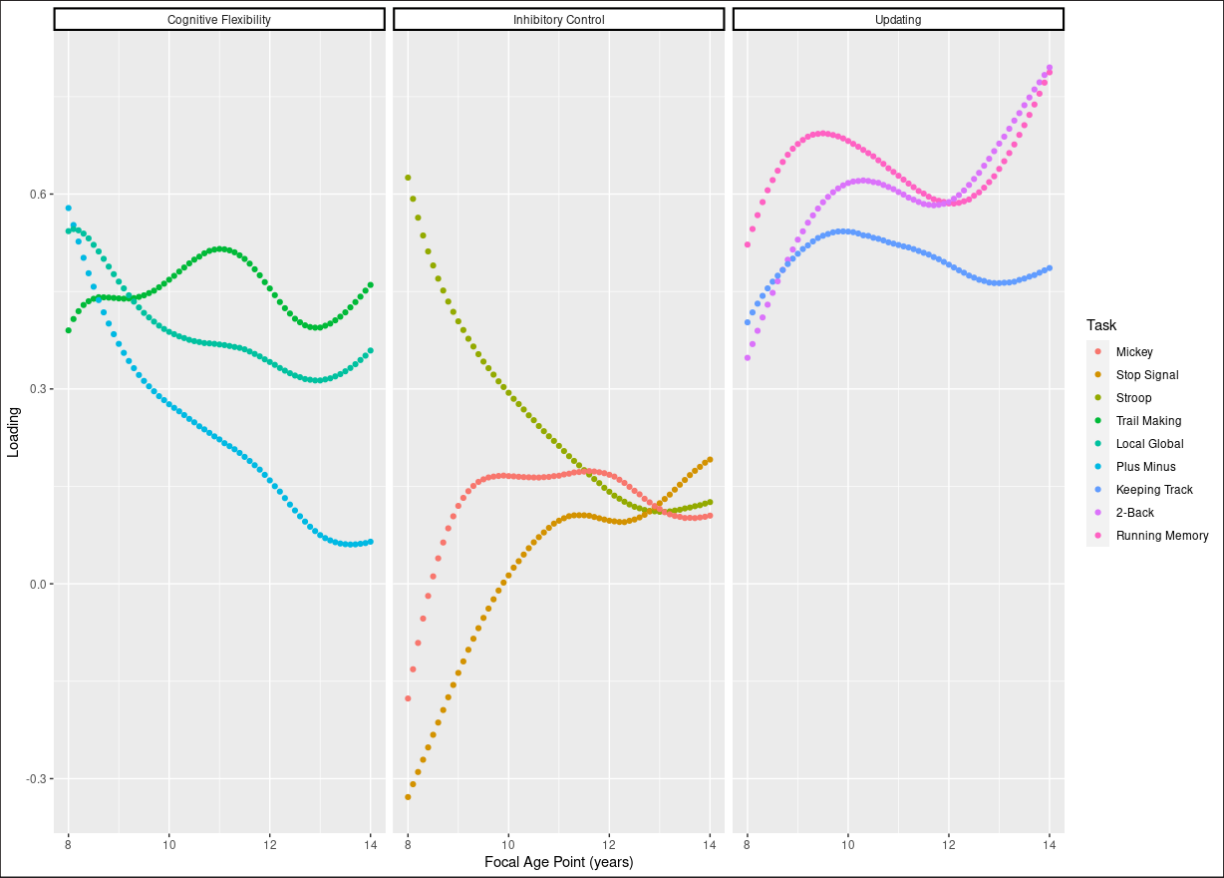
**Loadings of the 9 EF variables with age in the unidimensional models**.

**Figure 4:**
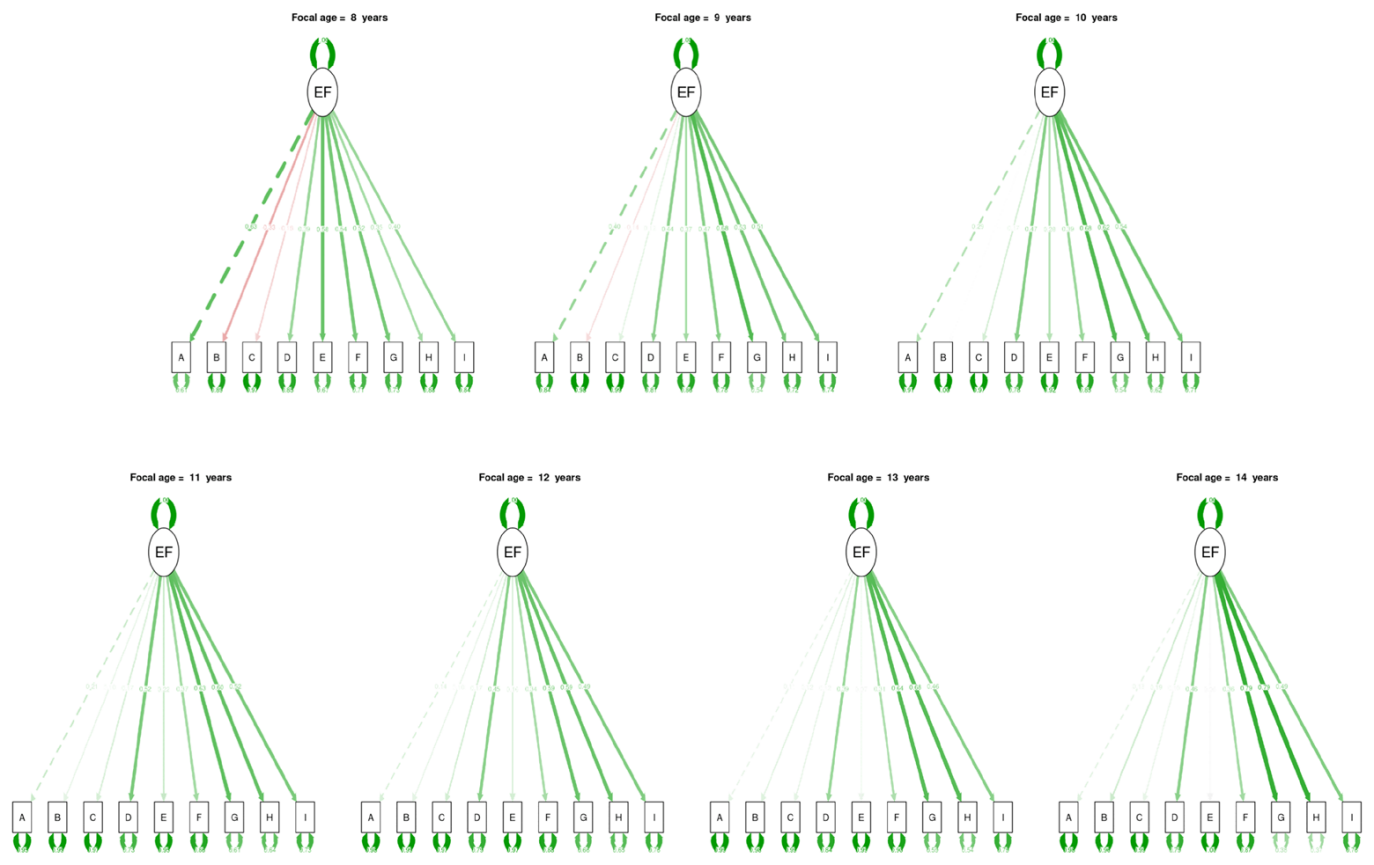
**Overview of the unidimensional latent model at different focal age points**. A = Stroop; B = Stop signal; C = Mickey; D = Trail making; E = Plus-minus; F = Local global; G = 2-back; H = Running memory; I = Keeping track. Green edges indicate positive loadings, red edges indicate negative loadings, and the thickness indicates the magnitude of these loadings.

We focus on this section on the loadings obtained for the unidimensional models (i.e., one latent factor defined by the 9 EF variables; Figures 3 and 4) which provides a simple comparison with the network analyses presented above. Indeed, the loadings represent the strength and direction of the relationships between the observed variables and the underlying latent EF, which is the common-EF for the unidimensional model. Of note, the findings obtained with the unidimensional models are highly similar to the findings obtained with the 3-factor models (see **Figure S5** in **Supplemental Materials**).

The loadings of different tasks do not contribute to common-EF in the same way during development, in line with the early differentiation reported above. In particular, the loadings of inhibition tasks are high at 8 years old but decrease drastically with age to stabilize around a low loading of 0.15 around age 12, reflecting the early differentiation of inhibition tasks from common-EF. On the other hand, the increasing loadings in updating tasks following 12 years of age indicate the increasing contribution of these tasks to common-EF, especially for the 2-Back and Running Memory tasks.

Similar to the findings reported in the network analyses, the loading patterns vary between different tasks of cognitive flexibility and inhibition, while the loading patterns are more homogeneous for updating tasks. Specifically, the Mickey and stop signal tasks both present negative loadings, meaning a negative relationship with common-EF, at early ages, in contrast to the Stroop task, which presents the highest positive loadings at 8 years old. This suggests that, initially, these inhibition tasks are inversely related to the latent variable, here the common EF. However, this effect could also be related to the poor fit of the model before 10 years of age.

Animated figures of all models are available online in **Supplemental materials (Figures S6–S9)**.

### Latent network models

**Figure 5:**
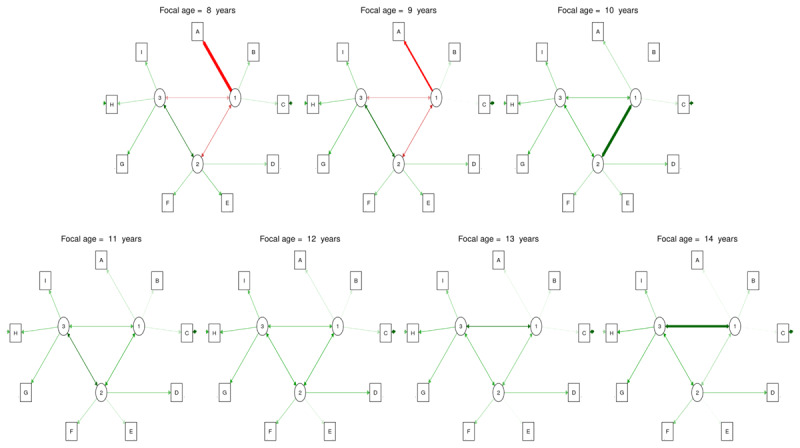
**Overview of the latent network model at different focal age points**. A = Stroop; B = Stop signal; C = Mickey; D = Trail making; E = Plus-minus; F = Local global; G = 2-back; H = Running memory; I = Keeping track. 1 = Inhibition; 2 = Cognitive flexibility; 3 = Updating. Green edges indicate positive weights, red edges indicate negative weights, and the thickness indicates the magnitude of these weights.

Latent network model analysis provides loadings for each latent variables, i.e., EF (see Figure 5). The factor loadings obtained from the latent network models are quite similar to the loadings obtained from the previous latent analysis: inhibition task loadings decrease through time, whereas loadings of updating tasks increase from 12 years of age. These similarities cross-validate the latent variable and latent network methods.

In addition to the task loadings, our latent network model analysis offers the unique opportunity to explore the network between the latent factors, i.e., the connectivity between EFs, and through development (see Figure 5). From 8 to 9 years old, the connections between inhibition and both cognitive flexibility and updating are low and negative but relatively stable. At the same period, the positive edge between updating and cognitive flexibility increases slightly. From 9.3 years old, inhibition and cognitive flexibility share a very strong and positive connection that starts to decrease slowly from 10.5 years old until 11 years old. Between 11 and 12.9 years old, the connection between EFs remains positive but weak with very few fluctuations. But from 12.9 years old, the connection between updating and inhibition starts to increase importantly and continues to do so until 14 years old.

Finally, the latent network model analysis indicates how the factor loadings contribute to the latent network. For example, between 8 and 9.1 years of age, the connections with inhibition are negative, while the loading of the Stroop task is high and negative. However, starting at 9.1 years old, all the connections of the EF network become and remain positive, which coincided with the switch to a positive loading from the Stroop task.

## Discussion

This study investigates the structure of executive functions across development and provides new findings at both theoretical and methodological levels. At the theoretical level, this study provides evidence of EF differentiation during development, with inhibition having a central role in childhood while updating smoothly gets this central place in early adolescence. At the methodological level, this study applies three complementary advanced analytic methods, including the recently developed latent network models combining network models and latent models.

A main strength of this study relies on the very consistent and similar findings provided by three different statistical approaches. The tasks with a substantial weight in the latent models were also the tasks with large and strong connections in the network models. The results reported in this study are likely independent from the specific method used to analyze the data. This is important as these methodologies are based on different assumptions. Indeed, latent (factorial) analyses are based on the hypothesis of a factorial organization while network model are *a priori* free of any hypothesis. Network models thus make it possible to assess the links between a variable of one EF with the variables of another EFs, while SEM cannot assess such links. On the contrary, SEM can address the general question of the factorial organization of EFs by directly testing and comparing different models with more or less latent factors. Finally, the latent network model analyses provide an additional level of information by assessing the links, without any *a priori*, between the theoretically-defined latent factors (here, the EFs).

At the EF level, these three complimentary methods revealed that the connections between the tasks and the ones between the latent variables decrease with age. This is particularly clear with the network analyses which resulted in a very integrated network with many inter- and intra-EF connections at early ages and a very sparse and segregated network for the oldest ages. For cognitive flexibility, a segregation at the intra-EF (fewer and weaker connections between cognitive flexibility tasks) and inter-EF (fewer and weaker connections between cognitive flexibility tasks and tasks of other EFs) levels was observed. Regarding inhibition, there was a specific pattern with initial negative loadings and connections of some tasks. These inter- and intra-EF connections then disappeared quite early in development. In the network models, inhibition was almost separated from the rest of the network, and, in the SEM, inhibition tasks had almost null loadings. Thus, there appears to be a very early dissociation of inhibition from other EFs, possibly reflecting the nested organization observed in adults (Karr et al., 2018). Finally, for updating, the intra-EF connections remained relatively stable with age. The inter-EF connections decreased a little during development but it remained the EF with the most connections with the other EFs in the oldest participants. This pattern is found in all models, where loadings and weights of edges between tasks or between latent variables increased in the last age groups. These findings are in line with previous studies demonstrating that inhibition becomes less central as individuals age and develop EFs, while updating becomes increasingly important in EF organization starting in adolescence and lasting until adulthood (Best et al., 2009; Huizinga et al., 2006; Isquith et al., 2004).

At the task level, the analyses provided complementary findings. For the updating tasks (*2-back, Running memory, Keeping track*), the three tasks followed the same trajectory with just a small gap in childhood (peak at 9.5 years for Keeping track and Running memory, peak at 10.5 years for 2-back). These very similar patterns suggest that the three updating tasks tap on similar processes, supporting a homogeneity of the measure. For the cognitive flexibility tasks (*Trail making, Plus-minus, Local global*), trajectories differed slightly, suggesting that the different tasks may tap on different cognitive flexibility processes. Of note, the use of Trail making as a measure of cognitive flexibility is debated (for a review: Sánchez-Cubillo et al., 2009), as this task requires also updating and inhibition (Brocki & Tillman, 2014; Chaytor et al., 2006; Chevalier et al., 2012; Miner & Ferraro, 1998). Finally, concerning the inhibition tasks (*Stroop, Stop signal, Mickey*), the three tasks initially had different properties, especially the Stroop task, which had opposite loadings and correlations compared to the two other inhibition tasks. These differences between tasks decreased with age until a homogenization around age 12. These differences may also be related to the different processes involved in the inhibition tasks : the Stroop task measures the interference control while the Mickey and stop signal tasks measure response inhibition (Diamond, 2013). Hence, these two inhibition components are likely initially distinct and become increasingly united and integrated with age.

Overall, our study supports the developmental differentiation of EFs, an hypothesis developed and applied in intelligence studies that suggest that the structure of a child’s development is unitary early in infancy but becomes more differentiated with age and the acquisition of cognitive abilities (Anderson & Nelson, 2005; Tucker-Drob, 2009). This differentiation, consistent with previous findings (Hartung et al., 2020; Karr et al., 2022; Younger et al., 2023), may also explain the different developmental trajectories of the three EFs reported in the literature (e.g., Best et al., 2009) in association with the segregation of brain networks with age (e.g., Baum et al., 2017). Our findings also suggest that there may be distinct sensitive periods for common-EF and each specific-EF components (Thompson & Steinbeis, 2020).

The findings of the three cutting-edge statistical approaches highlight their complementarity. Indeed, the network models allowed us to observe important connections between EF tasks that could not have been observed through latent or latent network models. We could also observe that some tasks were connected even though they are not theoretically related to the same EF. These connection can also be explained by the fact that some tasks tap on more than one EF, such as the Trail making and Local Global tasks designed to measure cognitive flexibility but which also solicit updating (Brocki & Tillman, 2014; Chevalier et al., 2012). These findings are consistent with previous network studies (Hartung et al., 2020; Younger et al., 2023) and raise issues regarding the *a priori* selection of tasks to define each EF latent variable. Future research should aim at understanding how the impurity of the tasks used to measure different EFs can contribute to a more refined understanding of EFs and their components. Overall, there is a need for research employing a range of EF tasks with both exploratory and confirmatory approaches, in order to enhance the accuracy of EF definitions, measurements, and therefore our understanding of EF organization.

The use of latent variable models gave us the opportunity to explore, for the first time, the relationships between EF constructs instead of EF tasks. The analyses revealed that correlations between EFs change with age, with an important coupling between inhibition and cognitive flexibility observed from 9.3 to 10.5 years old, and an important coupling between inhibition and updating observed from 12.9 years old. These EF-dependent changes raise the question of sensitive periods for EF coupling. Future research should explore the underlying mechanisms and developmental factors contributing to the observed age-related changes in the relationships between EFs. Studies identifying potential sensitive periods for EF interactions could provide insights into the timing of these interactions and inform intervention strategies for specific developmental stages.

Finally, the latent models allowed us to directly test the factorial organization of EFs (Karr et al., 2018; Miyake et al., 2000). We found that the seven theoretical models tested in this study provided very similar fit indices after the age of 10. This result is consistent with the low rates of model selection reported in Karr et al. (2018). In our analyses, the fit indices showed acceptable values after the age of 10, suggesting that model convergence was not an issue. Of note, model selection using fit indices was found to be associated with differences in the number of estimated parameters, more complex models usually fitting the data better than less complex ones (Morgan et al., 2015). Overall, our findings similarly supports models with different latent components related to different EFs and models with one common EF, in line with the unity/diversity of EFs, found at both behavioral (Miyake & Friedman, 2012) and neural (Engelhardt et al., 2019) levels. This finding could suggest that these factorial models should be considered from a hierarchical perspective as distinct but co-existing observational prisms, rather than as competing options.

Taken together, our findings demonstrates that EF differentiate throughout development. Of note, our analyses could not identify the ‘best’ theoretical factorial model that would outperform the other models, which raises the general issue of the relevance of latent models for EFs (Camerota et al., 2020). We do not think that latent models should be abandoned to analysis EF, but we consider that the use of a latent model should be carefully justified in line with the research question. If the research question regards the specificity of the EFs and their latent components, the latent models are highly relevant. If the research question is not focused on EF specificity but is more concerned with the relationships between EF tasks, then another model, for example a network model, might be more appropriate. Lastly, in examining the relationship between EFs and other areas of development, even though further research is necessary to fully comprehend the factorial organization of EFs, two approaches are available. The first approach is theory-driven, basing model construction on the literature in the domain, whereas the second is data-driven, using exploratory factor analysis or principal components analysis to arrange EF measures, potentially reducing them to fewer measurements.

Generally, the field of EFs can be complex due to the existence of numerous overlapping definitions across various disciplines (Nigg, 2017) and the EF measures are often non-pure (i.e., they involve other cognitive processes than just the targeted EF; Miyake et al., 2000). Additionally, latent model studies have unveiled the existence of subcomponents in each EF. For instance, a model of inhibition has demonstrated that interference control – the ability to resist interference from external stimuli and evaluated through visual matching tasks such as Stroop or Flanker tasks – and response inhibition – the ability to inhibit a prepotent motor response, typically measured through non-selective stopping tasks such as the stop signal or Go/No-go tasks – could be modeled as independent constructs, implying that they may be separate and functionally unrelated cognitive abilities (Tiego et al., 2018). It is also important to note that recent theories assert that EFs are developed to address specific tasks in our environment and are therefore dependent from the environment (Doebel, 2020). Interindividual differences and the factors that can influence them, including environment or culture (e.g., Roos et al., 2017), should then be taken into account.

Several methodological issues call for caution when interpreting our results. First, before the age of 10, the different models globally fitted poorly, in line with previous work (Karr et al., 2018), and thus should be interpreted with caution. This poor fit could be related to psychometry issues, measuring EFs in young participants is difficult and may be noisy and/or biased (Petersen et al., 2016). It can also be related to the high inter-individual variability in young ages, as observed in Younger et al. (2023). Further research using validated and development-sensitive measures, coupled with exploratory modeling approaches, are needed to produce more accurate and meaningful results in EF latent modeling before 10 years of age. Indeed, some of the results we observed could stem from how adaptable certain tasks are for specific age ranges. We cannot rule out the possibility of floor or ceiling effects influencing our results. This highlights the complexity of measuring EFs in developmental cohorts: while it is essential to keep the same protocol for comparisons, it may introduce age biases that could skew the results. Additionally, the various calculation methods of the dependent variables (reaction time vs. accuracy scores) could influence our results. Future studies should explore other calculation methods, such as inverse efficiency (Gärtner & Strobel, 2021) or drift rate (Yangüez et al., 2023) scores, to achieve better homogeneity in task measurements. Second, this study is based on a sample including twins. Pairs of participants of this sample therefore shares a significant common genetic part which we know impact differently the common-EF and the specific-EF components (Friedman et al., 2008). Then, this study focuses on ages 8 to 14, an important developmental window for EF development, but it would also be very interesting to investigate later stages of adolescence. The age range in the sample was relatively limited because the age sampling was very high. This dense sampling was necessary to investigate changes over very short age periods and detect fine-grained dynamics over the course of a year and also over the course of a month. However, this fine-grained sampling strategy resulted in a significant number of focal age points. While the issue of multiple testing is recognized, it remains an open issue in latent and network modeling literature. The fact that we estimated 61 networks using the same dataset exemplifies this issue of multiple testing, and raises questions about the robustness and interpretability of our results. Of note, classical guidelines recommend approximately 3–10 subjects per estimated parameter or variable for latent models (Bentler & Chou, 1987; Cattell, 1978) but, to our knowledge, there are no specific recommendations for network models. Finally, this study employed a cross-sectional approach, which, unlike a longitudinal approach with repeated measures, cannot track individual trajectories. To further validate and expand our findings, it is necessary to replicate them in an independent longitudinal sample of unrelated participants, spanning all stages of adolescence, while maintaining a comparable dense-sampling approach. In addition, while our results are supported by three different statistical methods, they are primarily based on descriptive analyses. Further inferential analyses are necessary for a definitive conclusion on the potential differentiation of EFs with age.

## Conclusion

In conclusion, this analysis study offers a comprehensive exploration of the structural organization of EFs during development, from 8 to 14 years of age, cross-validated with three complementary cutting-edge analytical approaches. Specifically, we observed an early differentiation of inhibition from the other EFs, followed by the differentiation of cognitive flexibility, while the role of updating increases in the structure of EFs with age. Importantly, major changes occurred within a single year, suggesting that future investigations should use narrow age ranges. While this study does not advocate for a specific factorial organization, our analyses provide support for the differentiation of EFs throughout development, a hypothesis which should be reevaluated in future inferential and independent studies.

## Data Accessibility Statement

Data are available upon request to the authors and with formal agreement of Prof. Harden & Tucker-Drob.

## Code availability statement

Code associated with the current submission is available at https://osf.io/2bzjc/.

## Additional File

The additional file for this article can be found as follows:

10.5334/joc.355.s1 Supplemental Materials. Appendix A1 and Figures S1–S10.
