# Supplementary material for: Latent Network Analysis of Executive Functions Across Development
Source: J Cogn. 2024 Apr 2;7(1):31. doi: 10.5334/joc.355 (PMC11012023; doi:10.5334/joc.355)
Supplement: Supplemental Materials. — Appendix A1 and Figures S1–S10. [file joc-7-1-355-s1.pdf]

Appendix A1: Presentation of degree, expected influence and closeness indices.

Figure S1: Degree of the 9 nodes in the network models across age.

Figure S2: Expected influence of the 9 nodes in the network models across age.

Figure S3: Closeness of the 9 nodes in the network models across age.

Figure S4: SRMR indices of the 7 latent models.

Figure S5: Loadings of the 9 EFs variables for the 3-factors and unidimensional models across ages.

Figure S6: Animated network model.

Figure S7: Animated unidimensional latent model.

Figure S8: Animated 3-factors latent model.

Figure S9: Animated latent variable network model.

Figure S10: Overview of the different models studied at each focal age point year with 12 variables.

Appendix A1: Presentation of degree, expected influence and closeness indices.

Degree indexes the number of connections for each node of the network, weighted by the size of these connections. Hubs correspond to nodes with the highest degrees. In contrary to degree which takes the *absolute* value of the weights, expected influence also takes into account the direction of the weight (negative or positive): it is calculated as the sum of both positive and negative weights between a node and all the other nodes in the network. Finally, closeness centrality is an index of how strongly a node is indirectly connected with the network. It is calculated as the inverse of the total length of all the shortest paths between the selected node and all other nodes in the network.

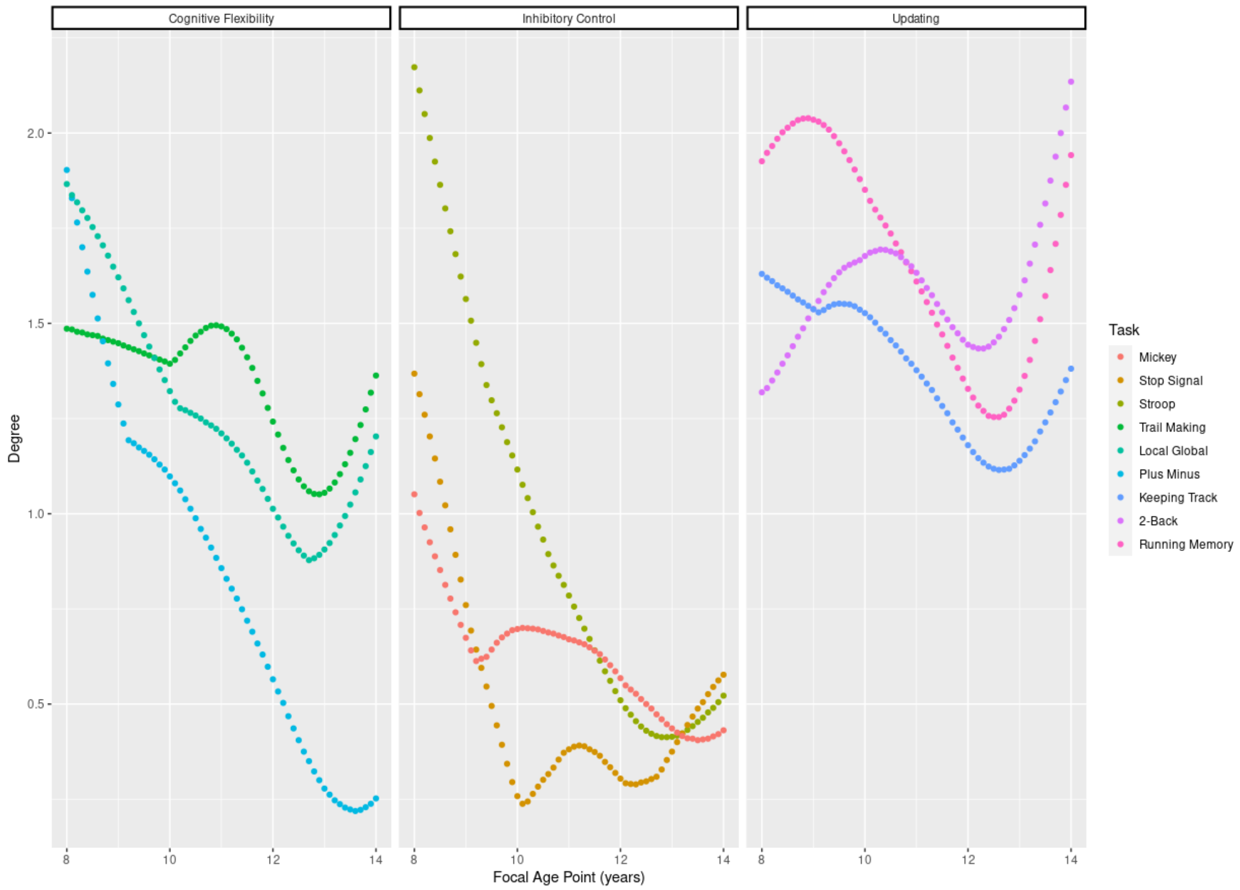

Figure S1: Degree of the 9 nodes in the network models across age.

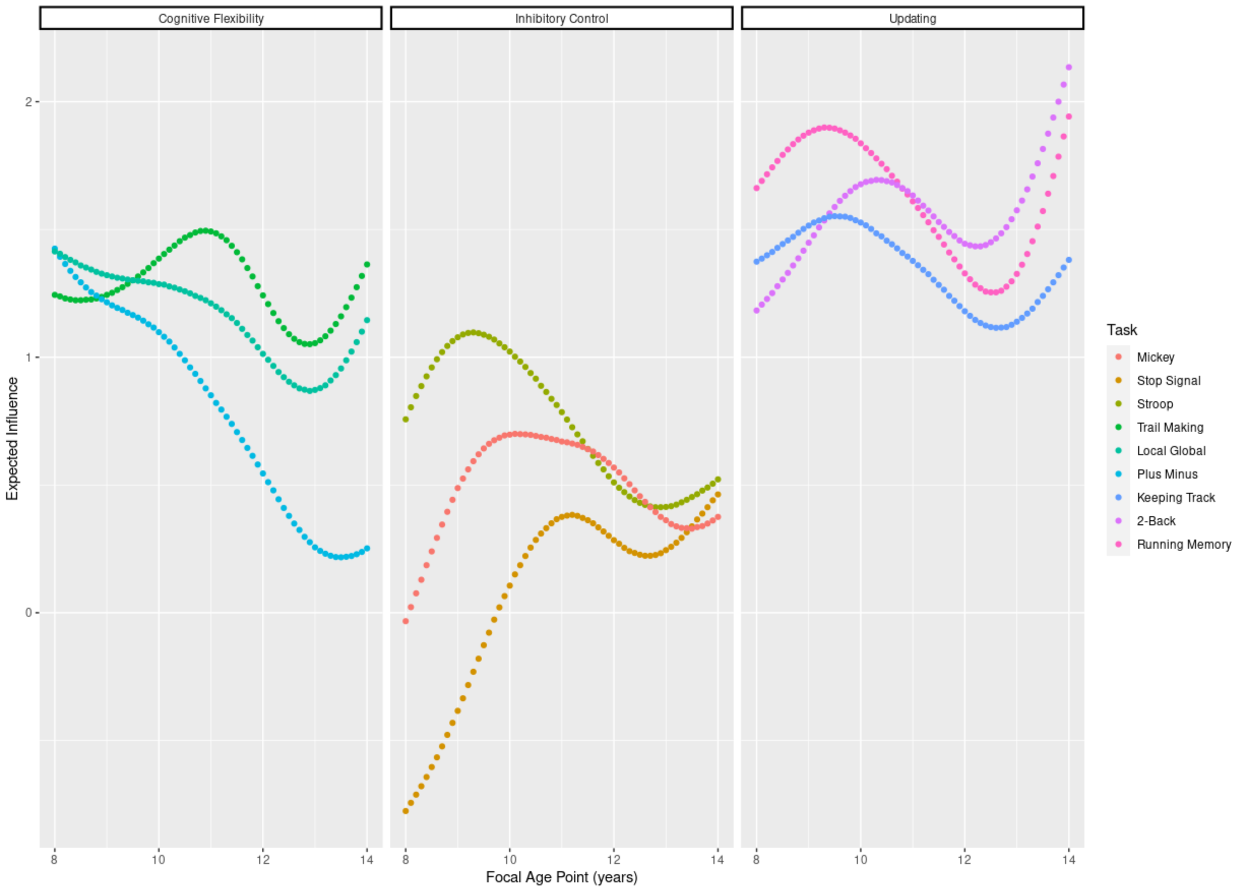

Figure S2: Expected influence of the 9 nodes in the network models across age.

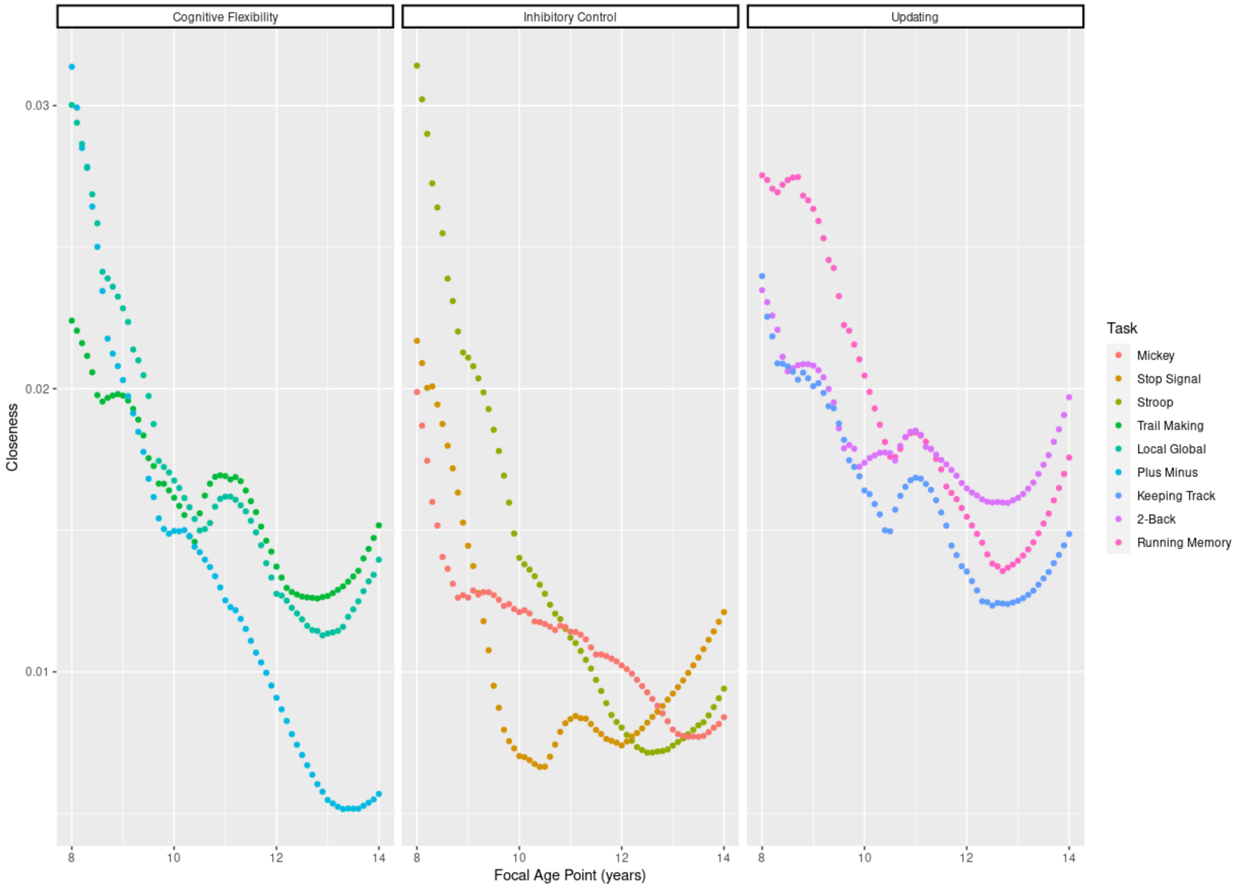

Figure S3: Closeness of the 9 nodes in the network models across age.

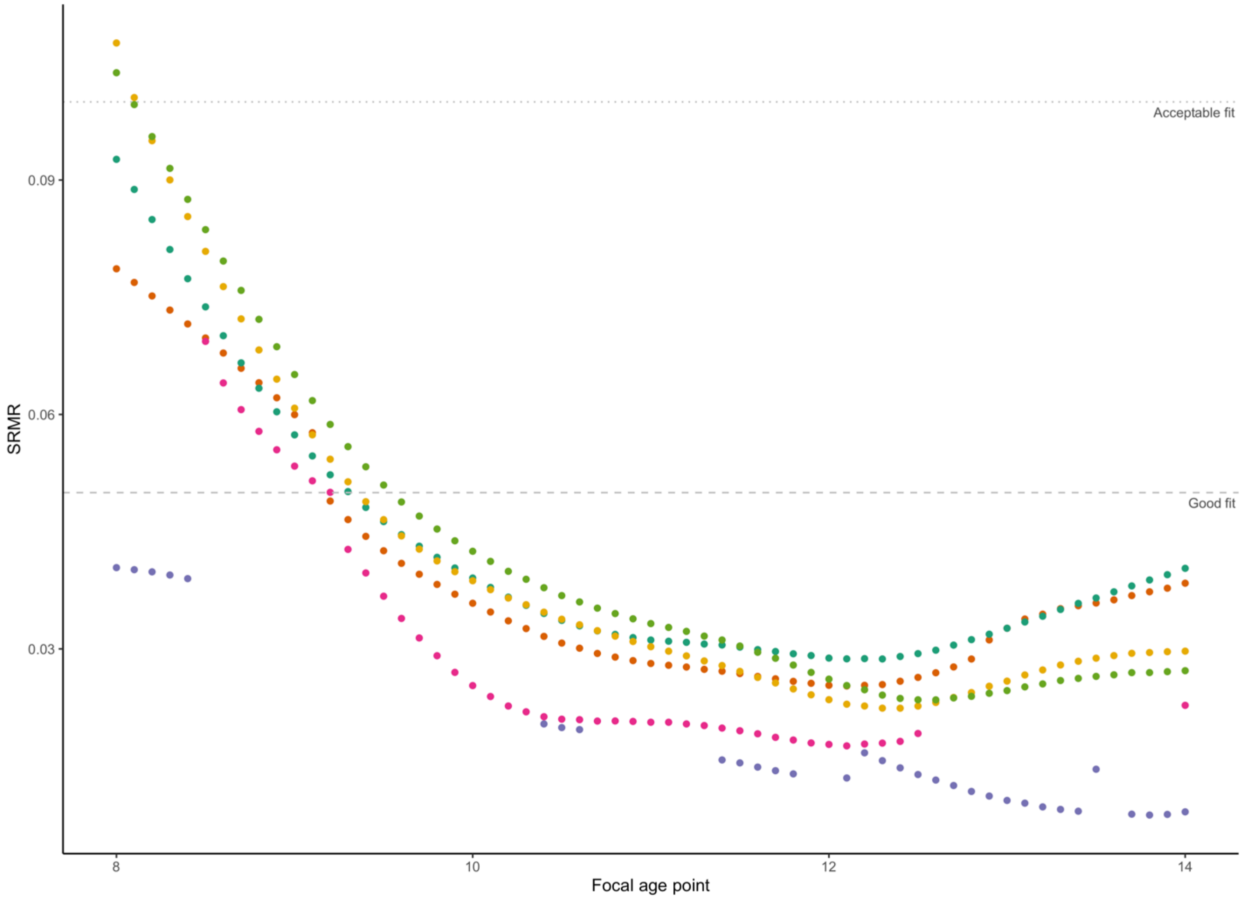

Figure S4: SRMR indices of the 7 latent models. Dot line corresponds to threshold for acceptable fit (SRMR < 0.10) and dashed line corresponds to threshold for good fit (SRMR < 0.05; Schermelleh-Engel et al., 2003).

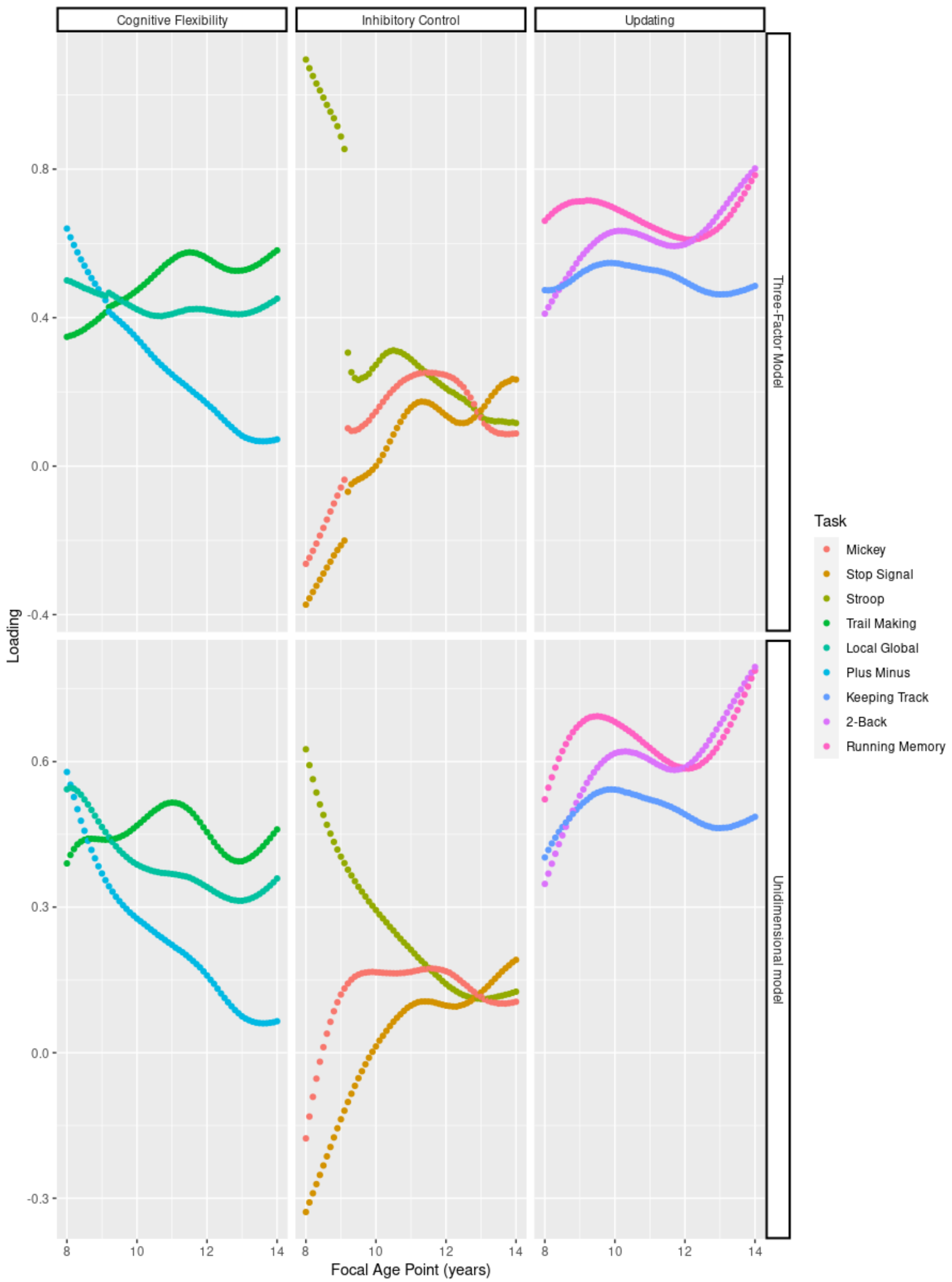

Figure S5: Loadings of the 9 EFs variables for the 3-factors (top) and unidimensional (bottom) models across ages.

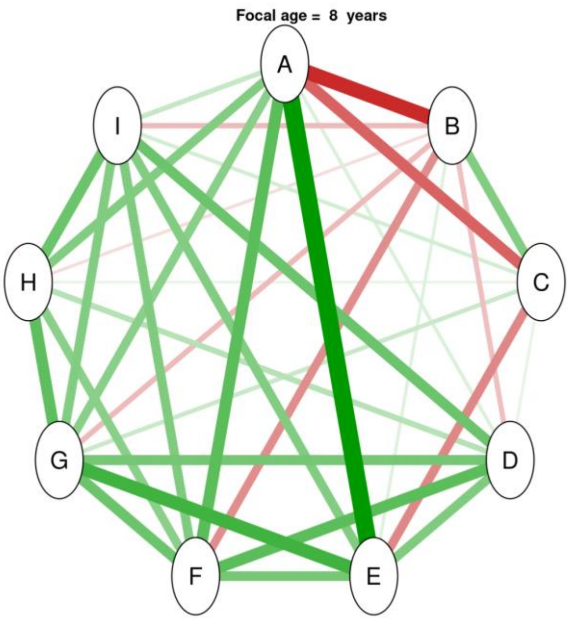

Figure S6: Animated network model. A = Stroop; B = Stop signal; C = Mickey; D = Trail making; E = Plus-minus; F = Local global; G = 2-back; H = Running memory; I = Keeping track. Green edges indicate positive weights, red edges indicate negative weights, and the thickness indicates the magnitude of these weights.

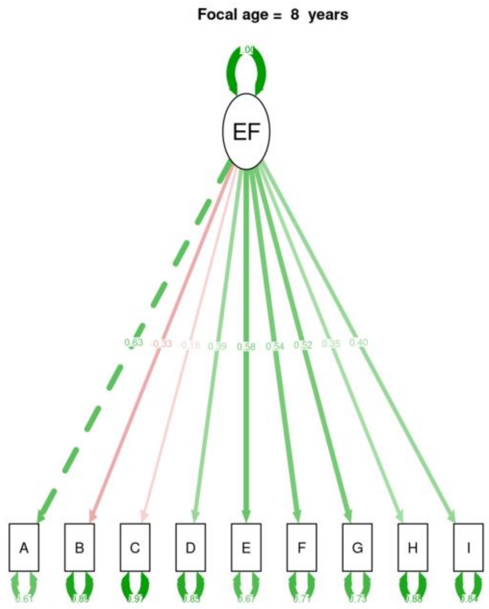

Figure S7: Animated unidimensional latent model. A = Stroop; B = Stop signal; C = Mickey; D = Trail making; E = Plus-minus; F = Local global; G = 2-back; H = Running memory; I = Keeping track. Green edges indicate positive loadings, red edges indicate negative loadings, and the thickness indicates the magnitude of these loadings.

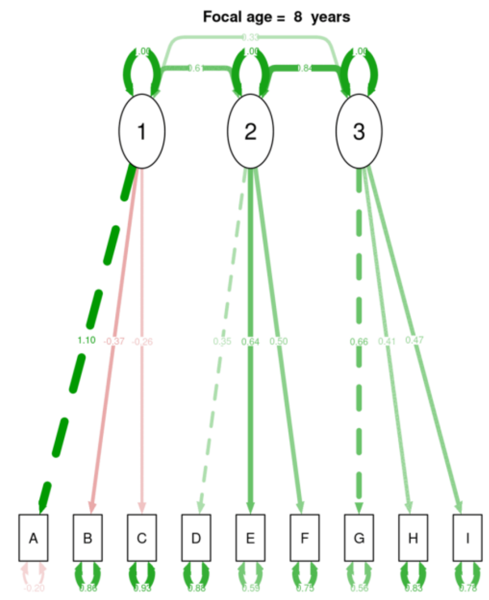

Figure S8: Animated 3-factors latent model. A = Stroop; B = Stop signal; C = Mickey; D = Trail making; E = Plus-minus; F = Local global; G = 2-back; H = Running memory; I = Keeping track. 1 = Inhibitory control; 2 = Cognitive flexibility; 3 = Working memory updating. Green edges indicate positive loadings, red edges indicate negative loadings, and the thickness indicates the magnitude of these loadings.

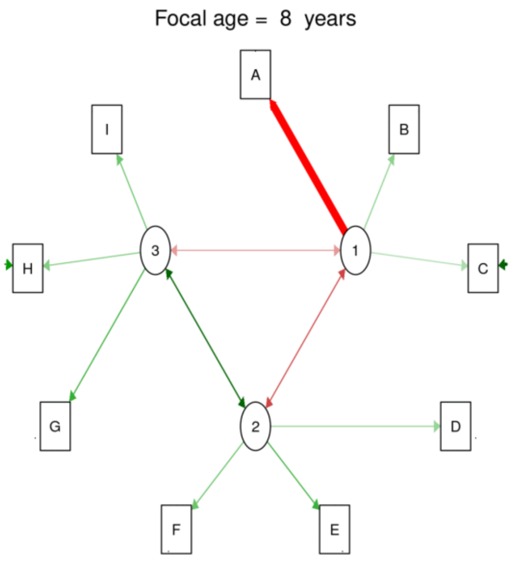

Figure S9: Animated latent variable network model. A = Stroop; B = Stop signal; C = Mickey; D = Trail making; E = Plus-minus; F = Local global; G = 2-back; H = Running memory; I = Keeping track. 1 = Inhibitory control; 2 = Cognitive flexibility; 3 = Working memory updating. Green edges indicate positive weights, red edges indicate negative weights, and the thickness indicates the magnitude of these weights.

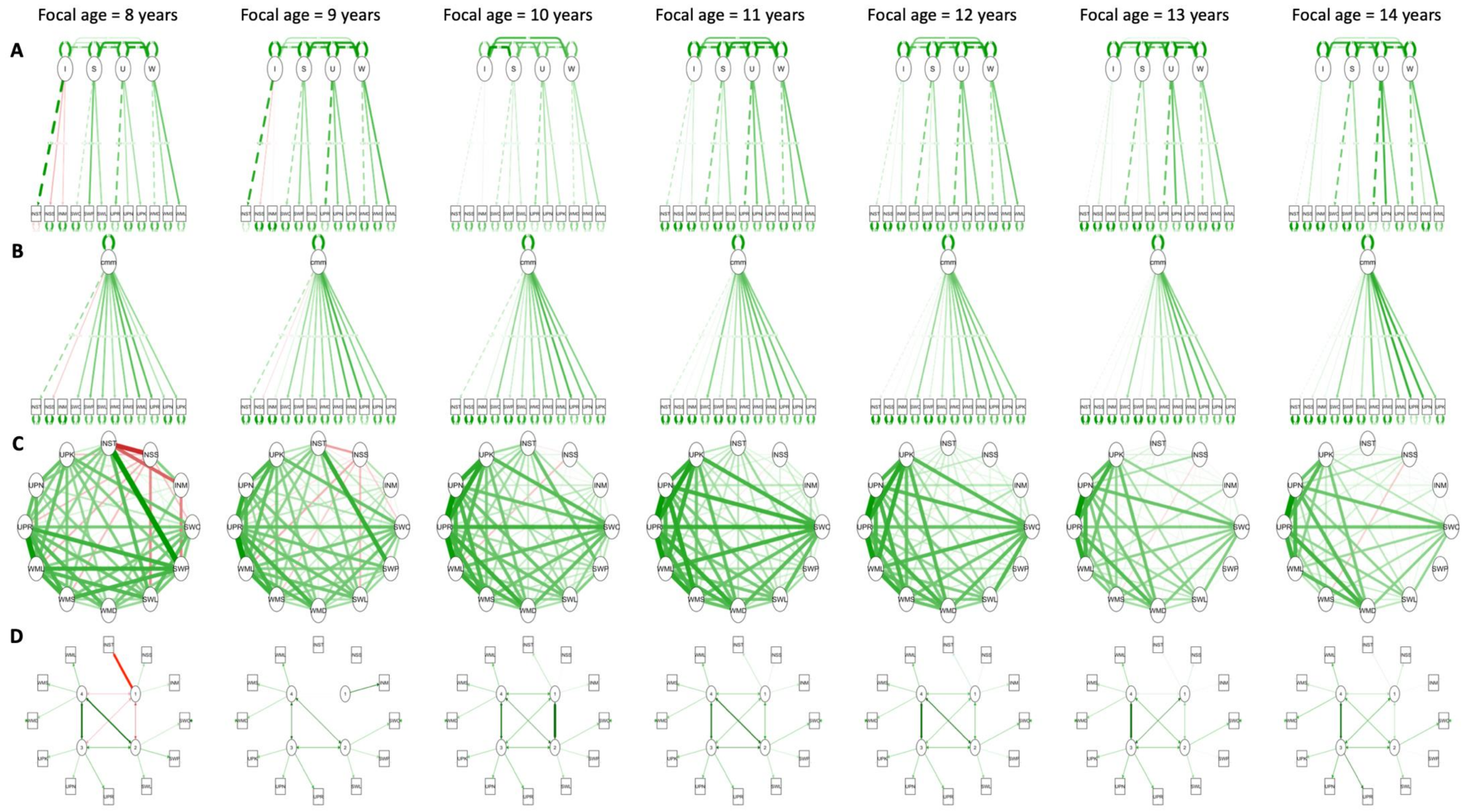

Figure S10: Overview of the different models studied at each focal age point year with 12 variables. Panel A: Three factors latent model; B: One factor latent model; C: Network model; D: Latent variable network model. INST = Stroop; INSS = Stop signal; INM = Mickey; SWC = Trail making; SWP = Plus-minus; SWL = Local global; UPN = 2-back; UPR = Running memory; UPK = Keeping track; WMD = Digit Span Backward; WMS = Symmetry Span; WML = Listen Recall. 1/I = Inhibitory control; 2/S = Cognitive flexibility; 3/U = Working memory updating; 4/W = Working memory. Green edges indicate positive loadings/weights, red edges indicate negative loadings/weights, and the thickness indicates the magnitude of these loadings/weights.
